# Next-generation sequencing of the mitochondrial genome of *Thamnaconus septentrionalis* Gunther, 1877 (Aluteridae: Thamnaconus) specimen collected in China

**DOI:** 10.1080/23802359.2021.1945501

**Published:** 2021-07-05

**Authors:** Bin Wang, Lei Wang, Anli Wang, Yutao Miao

**Affiliations:** a Guangdong Provincial Key Laboratory for Healthy and Safe Aquaculture, School of Life Sciences, South China Normal University, Guangzhou, China; b Institute of Modern Aquaculture Science and Engineering, School of Life Sciences, South China Normal University, Guangzhou, China

**Keywords:** *Thamnaconus septentrionalis*, mitogenome, phylogenetic tree

## Abstract

In this article, the complete 16,439 bp mitochondrial genome of *Thamnaconus septentrionalis* was determined from a specimen collected in China. It contains 13 protein-coding genes, 22 transfer RNA genes, 1 D-loop region, 2 rRNA genes, the base composition of A 27.74%, G 17.09%, T 26.04%, and C 29.13%, resulting in a G + C content of 46.22%. Phylogenetic analysis showed that *Thamnaconus septentrionalis* was the nearest sister to *Thamnaconus modestus*. These presented data will be significant for evolution relationships study among fish species.

The *Thamnaconus septentrionalis* belongs to the family Thamnaconus and the Order Aluteridae, and a marine bottom fish caught mainly in traditional fisheries (Kang et al. 2004). This fish is widely distributed in the Indo-West Pacific Ocean, ranging from the Korean Peninsula, Japan, and the China Sea to East Africa (Bian et al. 2020). At present, there is no report of the complete genome of *T. septentrionalis*, so we sequenced the mitochondrial genome of a Chinese specimen of *T. septentrionalis* to construct the taxonomy and phylogeny of this fish, providing more reference information relating to the superfamily of fishes (GenBank: MW485059).

A specimen of *T. septentrionalis* was collected from Weihai city (N37°30′36″, E122°6′36″) Shandong province, China, on 10 November 2020. It was preserved in 90% ethanol and stored at the Biological Herbarium (voucher no. SCNUFL05866), School of Life Sciences, South China Normal University. Samples were extracted from muscle tissue using the standard phenol-chloroform protocol. The paired-end DNA library with an insert size of 400 bp was constructed and sequenced by Illumina X-ten with 150 bp in reading length (Zhang et al. 2018). The de novo assembly of the mitochondrial genome was assembled by NOVOplasty (Dierckxsens et al. 2017). The complete mitochondrial genome of *T. septentrionalis* contains 13 protein-coding1 genes, 22 transfer RNA genes, 2 ribosomal RNA genes, 1 D-loop region, and the total length the 13 protein-coding genes is 11,433 bp. The mitogenome's whole base composition is A 27.9%, G 16.3%, T 24.8%, and C 31.0%., and C 29.13% with a slight C + G bias (46.22%) like other vertebrate mitochondrial genomes.

We performed a phylogenetic analysis of 14 affinity fishes based on 13 protein-coding gene sequences using the maximum-likelihood method implemented in the RAxML (Stamatakis 2014). In conclusion, *T. septentrionalis* was the nearest sister to *Thamnaconus modestus* (Figure 1)*.*

**Figure 1.**
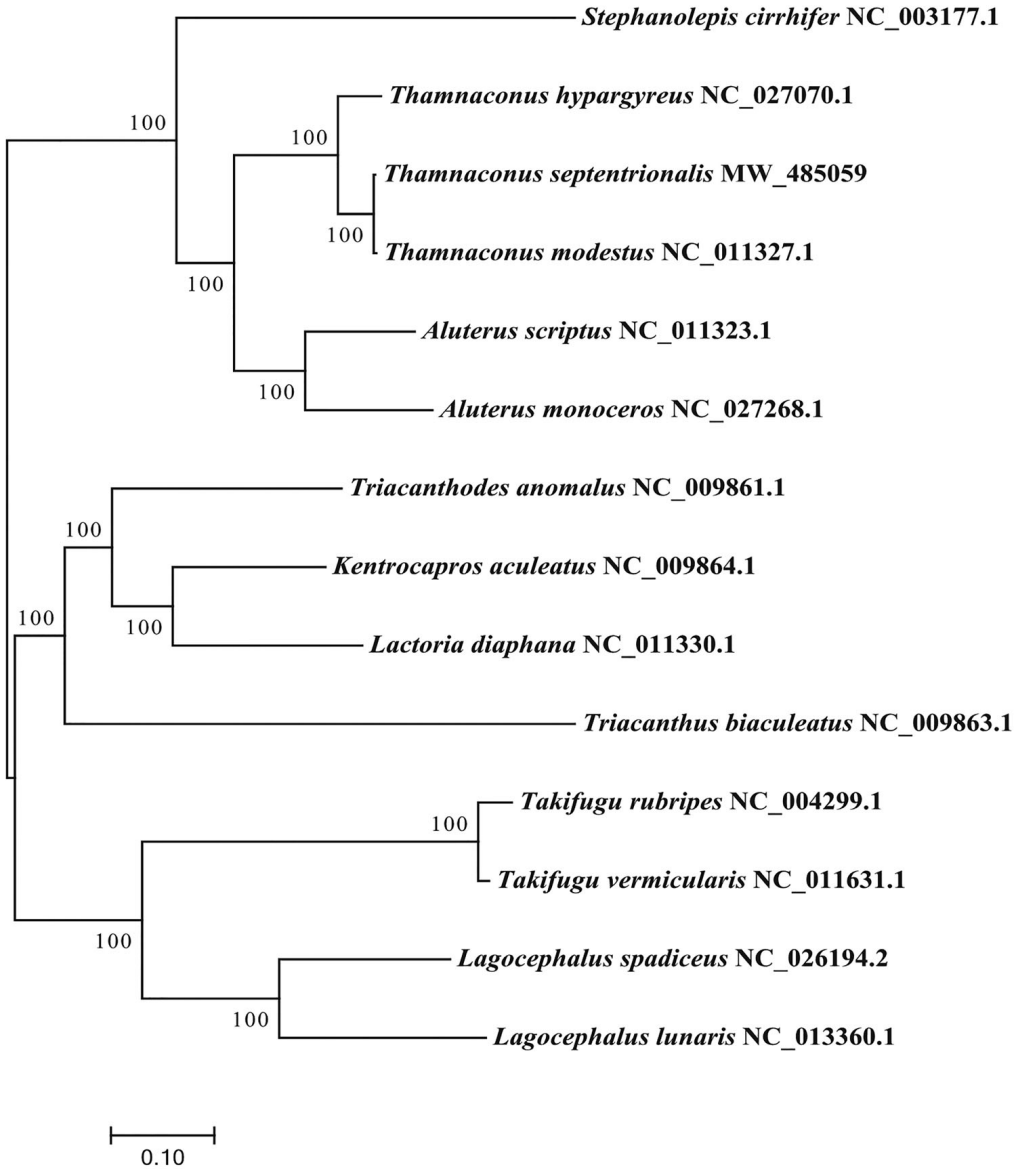
Phylogenetic tree generated using the maximum-likelihood method based on 13 protein-coding genes.

## Data Availability

The genome sequence data that support the findings of this study are openly available in GenBank of NCBI at https://www.ncbi.nlm.nih.gov/nuccore/MW485059 under the accession no. MW485059. The associated BioProject, SRA, and Bio-Sample numbers are PRJNA694825, SRR13528761, and SAMN17574947, respectively.
